# Identification of prognostic biomarkers in the CMTM family genes of human ovarian cancer through bioinformatics analysis and experimental verification

**DOI:** 10.3389/fgene.2022.918319

**Published:** 2022-08-30

**Authors:** Mengjun Zhang, Jialin Wang, Haodi Yue, Lindong Zhang

**Affiliations:** ^1^ Department of Gynecology, Third Affiliated Hospital of Zhengzhou University, Zhengzhou, China; ^2^ Department of Orthopedics, Capital Medical University Xuanwu Hospital, Beijing, China; ^3^ Department of Center for Clinical Single Cell Biomedicine, Henan Provincial People’s Hospital, Zhengzhou, China

**Keywords:** CMTM gene family, prognosis, ovarian cancer, immune microenvironment, biomarker

## Abstract

**Background:** Ovarian cancer (OV) is one of the most common gynecological malignancies worldwide, and its immunotherapy has considerable prospects. Multiple members of the CMTM family were aberrantly expressed in human cancers and controlled key malignant biological processes and immune regulation in cancer development. However, little is known about the function of this gene family in ovarian cancer, especially in terms of immunity.

**Methods:** GEPIA, Oncomine, HPA, Kaplan–Meier plotter, cBioPortal, GeneMANIA, and TIMER were used to analyze the differential gene expression, prognostic value, genetic alterations, and alterations in the immune microenvironment of the CMTM family in patients with ovarian cancer. Importantly, RT-qPCR was used to verify the gene expression of the CMTM family.

**Results:** CMTM1/3/4/6/7/8 showed abnormally high expression at the mRNA and protein levels in OV tissues based on the GEPIA and HPA databases. RT-qPCR showed that CMTM1/6/8 was highly expressed in ovarian cancer cell lines. IHC verified that CMTM8 is highly expressed in ovarian cancer tissues and is closely related to Ki-67. Survival analysis showed that high expression of CMTM1/2/3/5/8 can lead to a significant reduction in overall survival and progression-free survival. There were many types of genetic alterations in the CMTM family. Also, CMTM1/2/3/6 had a certain correlation with the changes in the immune microenvironment such as immune cell infiltration and immune checkpoint expression, which may be the potential mechanism of the CMTM family in ovarian cancer. IHC verified that CMTM6 is highly expressed in ovarian cancer tissues and is closely related to PD-L1.

**Conclusion:** This study confirmed that the CMTM family has abnormal expression in ovarian cancer and CMTM8 can be used as a biomarker for prognostic evaluation. Also, the CMTM family may be used as a potential target for immunotherapy based on the suppression of immune checkpoints.

## Introduction

Worldwide, 2,40,000 new cases of ovarian cancer, known as the “silent killer,” are diagnosed and 1,50,000 deaths occur each year. The diagnosis of ovarian cancer directly affects the prognosis (Ferlay et al., 2015). The 5-year survival rate for patients diagnosed with stage I is 90%. In patients whose disease has spread to nearby tissues or metastases far away, the 5-year survival rate drops to 80 and 25%, respectively (Vargas, 2014). With the development of genomics, the current trend in cancer treatment is precision medicine based on biomarkers. In recent decades, immunotherapy strategies have entered clinical practice in addition to gene-targeted therapies, and the treatment of ovarian cancer is undergoing a radical change (Xie et al., 2020). Yet, the use of immunotherapy remains controversial, and low immune response rates and immune resistance still need to be urgently addressed (Bogani et al., 2020). Therefore, the field of biomarker research not only needs to expand to the alterations of oncogenes or tumor suppressor genes but also more importantly, it is necessary to explore the dynamic alterations of epigenetics and immunology that occur in the tumor microenvironment.

CMTM1/2/3/4/5/6/7/8 belong to the CKLF-like MARVEL Transmembrane Domain (CMTM) gene family, which can be widely expressed on a variety of membrane structures and exert specific biological effects (Wu et al., 2020). CMTM members are located on different chromosomes: CMTM1-4 at 16q22.1, CMTM5 at 14q11.2, and CMTM6-8 at 3p22.3.2. The genes of the CMTM family form the abovementioned three chromosomes, and many key tumor suppressor genes are located in chromosome 3p22.3.2 (Han et al., 2003; Wu et al., 2020). The structure and function of the CMTM family are similar to those of the classical chemokine and transmembrane 4 superfamily (TM4SF), with different members having different RNA splicing forms and further forming several isoforms. Among them, CMTM1 contains a C-C motif and has high homology with chemokines, while CMTM8 shares 39.3% amino acid similarity with TM4SF11, and CMTM2-7 has biological properties between chemokines and TM4SF2 (Sánchez-Pulido et al., 2002; Wu et al., 2019; Duan et al., 2020). The members of the CMTM gene family have different chromosomal locations, structures, and biological functions, and these differences also lead to their different functions in physiology and pathology. To date, members of the CMTM family have been found to play regulatory roles in a variety of diseases, including autoimmunity, multiple malignancies, and cardiovascular disease (Hu et al., 2013; Delic et al., 2015; Cai et al., 2017; Song et al., 2021). Numerous studies have confirmed that the CMTM family plays an important role in tumorigenesis. On the one hand, CMTM2/3/5/7/8 was involved in the regulation of cancerous processes in several types of malignancies, such as cervical cancer, hepatocellular carcinoma, breast cancer, colorectal cancer, gastric cancer, and non-small-cell lung cancer (Guo et al., 2009; Li et al., 2011; Su et al., 2014; Huang et al., 2019; Wu et al., 2019; Guo et al., 2020). On the other hand, CMTM1 and 6 may function as oncogenes in glioma, non-small-cell lung cancer, gastric cancer, and liver cancer and are intimately interrelated with immunity, especially to the immune checkpoint PD-L1 (Li et al., 2020). The CMTM family genes not only play a pivotal role in the malignant biological behavior of tumors and anti-tumor immunity but also are closely related to the clinical characteristics of tumors, chemotherapy resistance, and prognosis (Supplementary Table S1 for details). However, up to now, there is no relevant research on the CMTM gene family in ovarian cancer.

As the clinical benefit of immuno-oncology research has attracted much attention, there is a consensus that the role of the immune system in the development of tumors is significant. Specifically, immune factors, infiltrating immune cells, and immune checkpoints are also considered to play a crucial role in carcinogenesis and have been used as targets for cancer therapies such as anti-PD-1/PD-L1 therapies (Matsuzaki et al., 2010). It is promising that the role of several members of the CMTM family in immunobiology has been revealed. CMTM6, an important regulator of PD-L1 protein, has been shown to play a vital role in tumorigenesis and progression (Galon et al., 2013). In addition, CMTM4 may also serve as an alternate regulator of stabilizing PD-L1 protein (Mezzadra et al., 2017). The detailed immune-related functions and mechanisms of the CMTM gene family have not yet been fully elucidated, especially in ovarian cancer, so further exploration is needed to maximize the benefits for patients with ovarian cancer.

We are the initial study to investigate the crucial role of CMTMs in poor prognosis and altered immune microenvironment in ovarian cancer. The study can be seen in Supplementary Figure S1 of the schematic diagram. Here, we first investigated the aberrant expression of CMTMs in pan-cancer in-depth and then explored the differential expression of CMTMs in ovarian cancer and the impact on poor prognosis at the nucleic acid level and protein level, respectively. In parallel, the RT-qPCR was performed to validate the results. Then, it is exciting to find a close correlation between CMTM expression and the tumor immune microenvironment (including immune cell infiltration, immune checkpoints, and chemokines) of the tumor. Finally, we sought to explore the contribution of CMTMs to poor survival outcomes in ovarian cancer patients through analyzing alterations in the immune microenvironment. In conclusion, this study sought to explore the potential of CMTMs as new immune-related prognostic biomarkers in OV patients, which may open a new avenue for the combination of immunotherapy and gene therapy in OV patients.

## Materials and methods

### Analysis based on GEPIA and TCGA

Gene Expression Profiling Interactive Analysis (GEPIA, http://gepia.cancer-pku.cn/index.html) is a newly developed huge resource database used to detail the RNA sequencing expression data of a variety of tumor and normal samples from the Cancer Genome Atlas (TCGA). Pan-cancer expression analysis, differential expression analysis of tumor and normal tissues, and clinical characteristics correlation analysis were performed through the GEPIA and TCGA databases. The eight members of the CMTM gene family were analyzed from the abovementioned multiple perspectives.

### ONCOMINE analysis

The ONCOMINE database (https://www.oncomine.org/resource/main.html) is a freely accessible online cancer microarray database that provides genome-wide expression analysis. It was used to determine the differences in transcript levels of the CMTM family between different cancer tissues and normal control tissues. In this study, parameters *p* < 0.01, fold change >1.5, gene class significance threshold: 10%, and data type: mRNA.

### Survival analysis based on Kaplan–Meier Plotter

Kaplan–Meier Plotter (http://kmplot.com/analysis/) is an online tool that contains survival information for breast cancer (*n* = 6234), stomach cancer (*n* = 1440), lung cancer (*n* = 3452), and ovarian cancer (*n* = 2190). This tool is mainly based on gene expression information and clinical survival information from the GEO and TCGA database to verify the impact of identified biomarker genes on the prognosis of patients. In this study, the Kaplan–Meier Plotter was used to evaluate the impact of the eight different CMTM family members on the prognosis of patients with ovarian cancer. According to the best cut-off value of mRNA expression of the eight different CMTM family members, patients with ovarian cancer were divided into the high-expression group and low-expression group. The relevant risk information included HR (Hazard Ratio), 95% CI (Confidence Interval), and *p* value. We considered the statistically significant difference when the *p* value is < 0.05.

### Correlation analysis of gene mutation based on cBioPortal

The cBioPortal (www.cbioportal.org) is a comprehensive resource data site containing genomic and clinical data that allows multidimensional visualization of genomic data in complex cancers. In this study, the mutation frequencies of eight genes in the CMTM family were explored, and the Kaplan–Meier method was used to graphically show the relationship between CMTM mutations and overall survival (OS) and disease-free survival (DFS). *p* value < 0.05 was considered statistically significant.

### HPA-based immunohistochemistry

The Human Protein Atlas (http://www.prote inatlas.org) is an immunohistochemical database used to validate genes with prognostic values. The differential expression of CMTMs between ovarian cancer tissues and control normal tissues at the protein level was explored.

### GO and KEGG analysis

Gene Ontology (GO) and the Kyoto Encyclopedia of Genes and Genomes (KEGG) were used to annotate and visualize gene functions and enrichment pathways. Gene ontology analysis focuses on three domains: the biological process (BP), cellular component (CC), and molecular function (MF), and such analysis is often used to predict the functional role of CMTM families and associated genes. KEGG analysis allows the exploration of pathways associated with CMTM families. The related genes whose expression was correlated with CMTM family genes and whose Pearson correlation coefficient was greater than 0.3 were included in GO and KEGG analysis. Only terms with *p* value <0.05 were considered significant.

### Co-expressed gene interaction network based on GeneMANIA

GeneMANIA (http://www.genemania.org) is a resource-rich website containing gene information, analyzing gene lists and prioritizing genes for functional assays with high accuracy of the prediction algorithm. Gene co-expression analysis was performed to explore specific genes that have co-expression relationships or interaction relationships with CMTM gene family members through this database.

### Analysis of immune infiltration and immune checkpoint based on the TIMER database

Tumor Immune Estimation Resource (TIMER, https://cistrome.shinyapps.io/timer) is powerful tumor immunology and genetics-related database. It is an online data site for assessing the infiltration of different immune cells and their clinical impact. In this study, via the TIMER database, we evaluated the association of CMTM expression with infiltration of six different immune cell types (B cells, CD4 + T cells, CD8 + T cells, macrophages, neutrophils, and dendritic cells). In addition, the correlation between CMTM family genes and immune checkpoints was assessed and the influence of the expression level of CMTM gene family members on patient survival has also been explored. At last, the role played by copy number variants in this gene family in tumor immunity was also explored.

### Patients and clinical tissue samples

In this study, we collected 98 paraffin-embedded ovarian cancer tissue samples and 12 paraffin-embedded normal ovarian tissue samples from gynecological surgery patients in the Affiliated Tumor Hospital of Harbin Medical University from January 2016 to May 2021. This study was reviewed and approved by the Ethics Committee of Harbin Medical University Cancer Hospital. Written informed consent was obtained from all patients. Supplementary Table S2 summarizes the clinicopathological characteristics of the study participants.

### Cell culture

Human ovarian cancer cells (SKOV-3 and A2780) and human ovarian cells (IOSE-80) were purchased from The Cell Bank of Type Culture Collection of Chinese Academy of Sciences. All cells were grown in incubators at 37°C and 5% carbon dioxide and cultured using DMEM medium supplemented with 10% FBS (both from Thermo Fisher Scientific Inc.).

### RT-qPCR

Total RNA was isolated from corresponding cell lines. Then, the concentration and purity of total RNA were determined by using a NanoDrop One spectrophotometer (Thermo Fisher Scientific), and reverse transcription was performed to obtain cDNA (Novoprotein). Finally, the expression level of CMTM1-8 was determined by RT-qPCR using NovoStart SYBR qPCR SuperMix Plus (Novoprotein). The primer sequences of GAPDH and CMTM1-8 are shown in Table 1. The thermal cycling conditions were as follows: initial denaturation at 95°C for 10 min, denaturation at 95°C for 10 s, and annealing and extension at 60°C for 30 s, for a total of 40 cycles. The relative expression level of the gene is calculated by the 2^−ΔΔ^CT method. The average values of gene expression levels are compared by the *t*-test. *p* < 0.05 was considered statistically significant.

**TABLE 1:** Sequences of primers used for qRT-PCR.

Gene	Primer sequence (5′-3′)
CMTM1-F	CCTTCACCACTTGCTGACCT
CMTM1-R	ATGCAACACACGATTACCGC
CMTM2-F	AAGAAGGACGGTAAGGAGCCA
CMTM2-R	GCACCGCCTTTTGAGGTTTG
CMTM3-F	CGAGTCGGGTCTCTCATTCAT
CMTM3-R	CCCTGCCACTTGTCATTCAG
CMTM4-F	TTCAATCGTACTGGCTGCTTT
CMTM4-R	CCAGGAATGTGTTCACTGCATA
CMTM5-F	GCTGCTTTTGTTTTTGGCATCA
CMTM5-R	TGCCATCTCAGTCCGGTAGA
CMTM6-F	TTTCCACACATGACAGGACTTC
CMTM6-R	GGCTTCAGCCCTAGTGGTAT
CMTM7-F	GCGCCTACAGCTACTTTGAA
CMTM7-R	AAACCAAAGATCGACAGGGG
CMTM8-F	GCTGGTATGGACGCTTATTGC
CMTM8-R	GGTGAGGACCCAGTAAAATACAG
GAPDH-F	CAAGGTCATCCATGACAACTTTG
GAPDH-R	GTCCACCACCCTGTTGCTGTAG

### Immunohistochemistry (IHC)

Paraffin-embedded tissue sections were prepared as 4 µm tissue sections. Tissue sections were then dewaxed in xylene and hydrated in an ethanol gradient. After rinsing with distilled water, the activity of endogenous peroxidase was blocked with hydrogen oxide solution. Sections were autoclaved for 4 min (121°C). Rabbit anti-human monoclonal antibodies specific for CMTM8 and Ki-67 (1:200 dilution; Abcam, Cambridge) were used in normal ovarian tissue sections, ovarian cancer tissue sections, and ovarian cancer tissue sections of different FIGO stages, respectively, at 4°C and incubated overnight. The same ovarian cancer tissue sections were incubated with specific rabbit anti-human monoclonal antibodies for CMTM6 and PD-L1 (1:200 dilution; Abcam, Cambridge) overnight at 4°C to verify the relationship between their expression levels. After three washes in PBS, goat anti-rabbit secondary antibody (1:5000 dilution; Abcam) was incubated for 1 h at room temperature on a shaker. Sections were dehydrated in graded ethanol series and sealed with neutral resin. Finally, the percentage and staining intensity of positively stained cells were observed under a light microscope.

### Statistical analysis

The R software (v.3.6.1 version) was used to perform statistical data analysis. Survival analysis and correlation analysis of clinical characteristics were performed based on databases such as TCGA. Survival analysis was performed by the Kaplan-Meier method. The correlation analysis of clinical characteristics was performed by the Wilcoxon rank sum test. The correlation analysis between gene expression and immune infiltration was performed by Spearman’s method.

## Results

### Aberrant expression of the CMTM family in ovarian cancer patients

**FIGURE 1:**
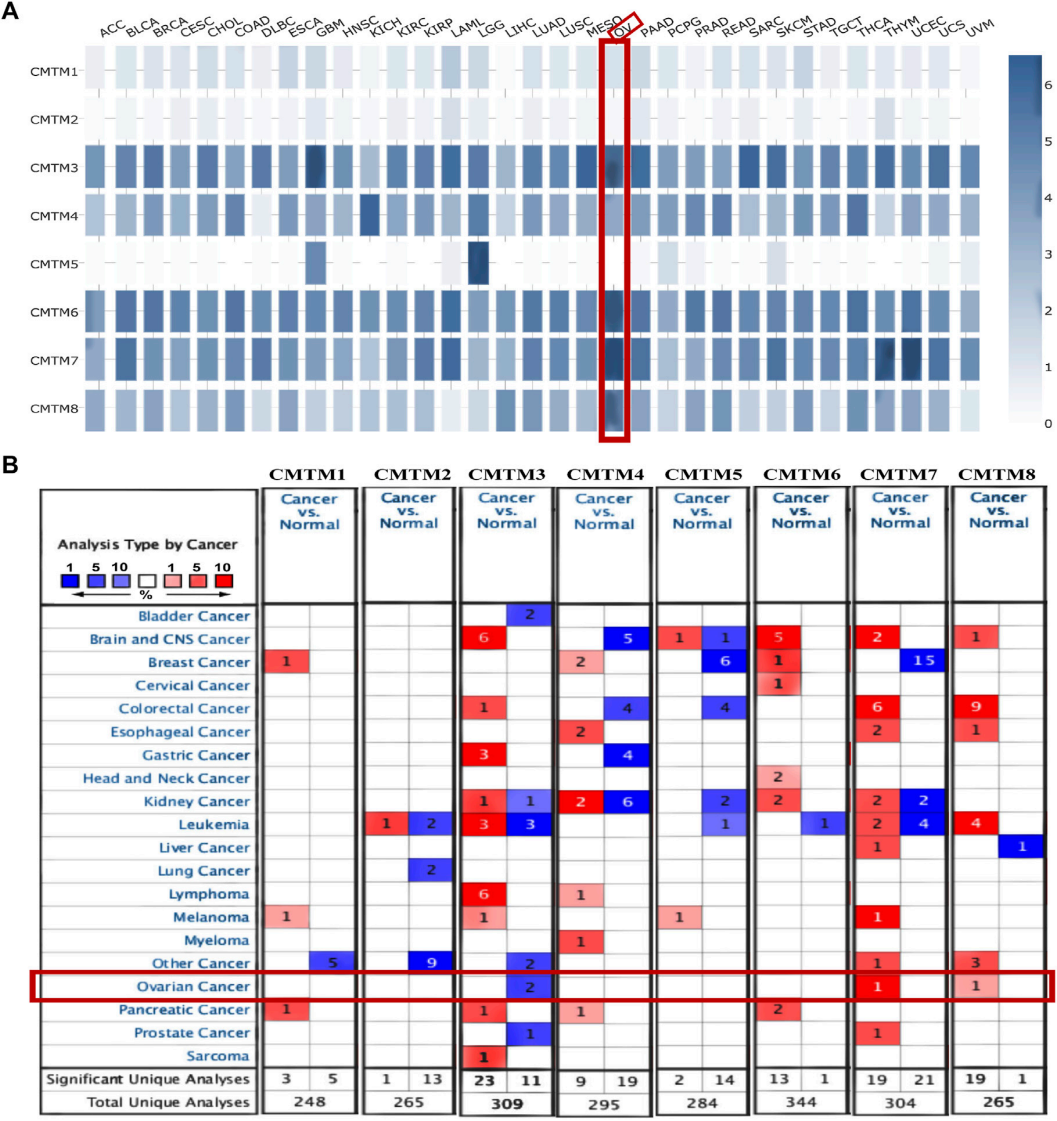
Transcriptional expression of CMTM family gene in pan-cancer. **(A)** The expression of CMTMs in different types of tumor tissues in the GEPIA database. The shade of the blue square represents the level of gene expression. The darker the blue, the higher the gene expression level. Ovarian cancer was specifically selected with a red line frame. The expression level of CMTM3/4/6/7/8 was relatively high in ovarian cancer. **(B)** The mRNA levels of each member of the CMTM family in different types of cancer and the number of related studies based on the ONCOMINE database. A red square represents a relatively high expression of the gene in a relevant study, a blue square represents a relatively low expression of the gene in a relevant study, and the number in the square represents the number of relevant studies. For example, in breast cancer, there are 15 relevant studies all showing a meaningful relative expression of CMTM7. Statistical significance parameters: *p* < 0.01, fold change >1.5, gene class significance threshold: 10%, data type: mRNA.

**FIGURE 2:**
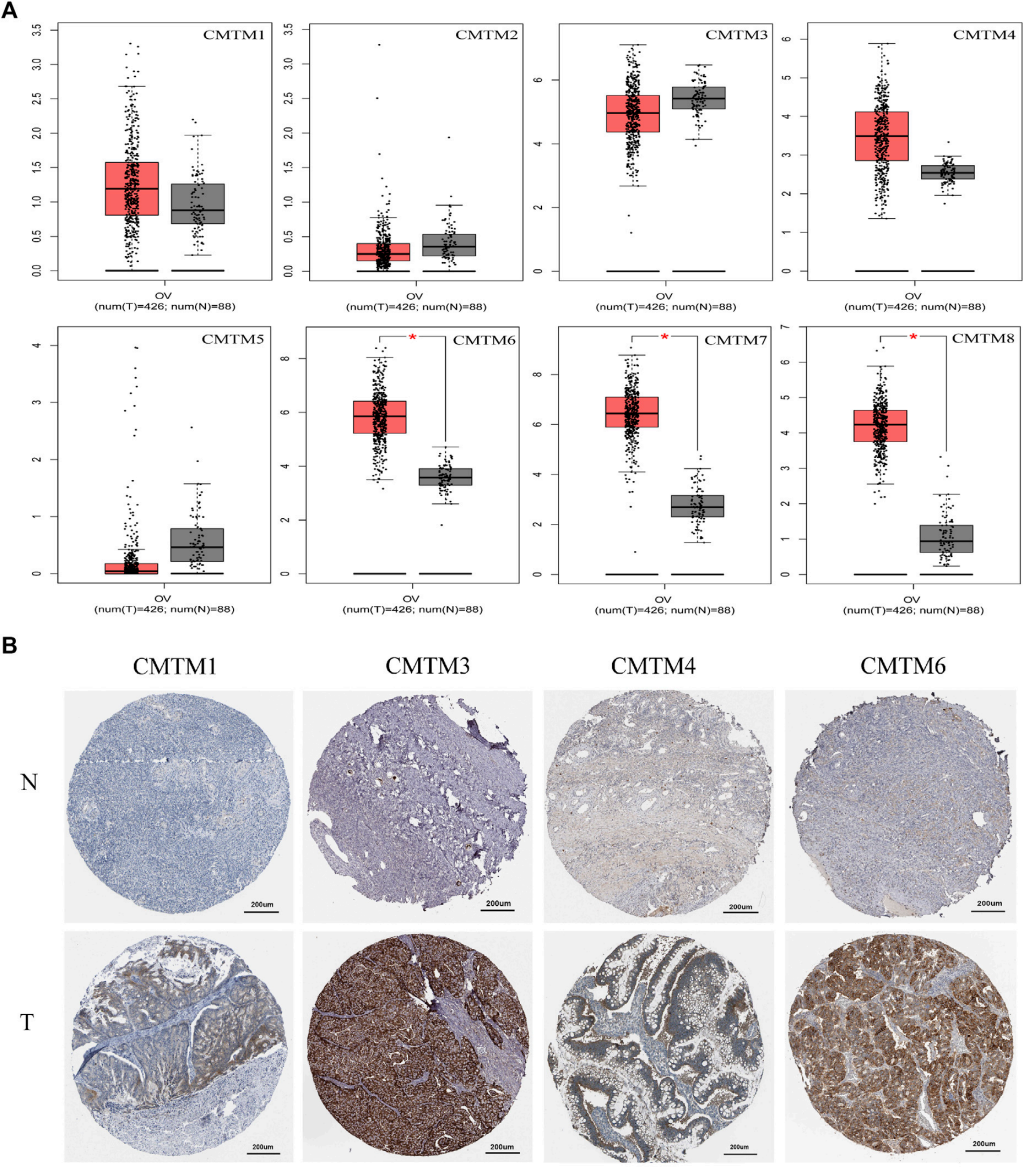
The expression levels of each member of the CMTM family at the mRNA and protein levels in ovarian cancer tissue and normal ovarian tissue. **(A)** Box plot of mRNA expression of different CMTM family members in OV tissue (*n* = 426) and normal ovarian tissue (*n* = 88) based on the GEPIA database. Compared with normal samples, the mRNA expression of CMTM6/7/8 was found to be relatively high in OV tissue at the mRNA level (*p < 0.05). **(B)** Immunohistochemical stained sections of CMTM1/3/4/6 in OV tissue and normal ovarian tissue based on HPA database. It can be seen that the higher staining intensity in OV tissue means that CMTM1/3/4/6 was relatively highly expressed at the protein level in ovarian cancer. The ID of the sample and the antibody used were recorded. CMTM1 (ID: 2713; ID: 1310; Antibody: HPA13801), CMTM3 (ID: 2004; ID: 3116; Antibody: HPA072695), CMTM4 (ID: 2159; ID: 1844; Antibody: HPA023890, HPA014704), CMTM6 (ID: 2713; ID: 2347; Antibody: HPA026980).

**FIGURE 3:**
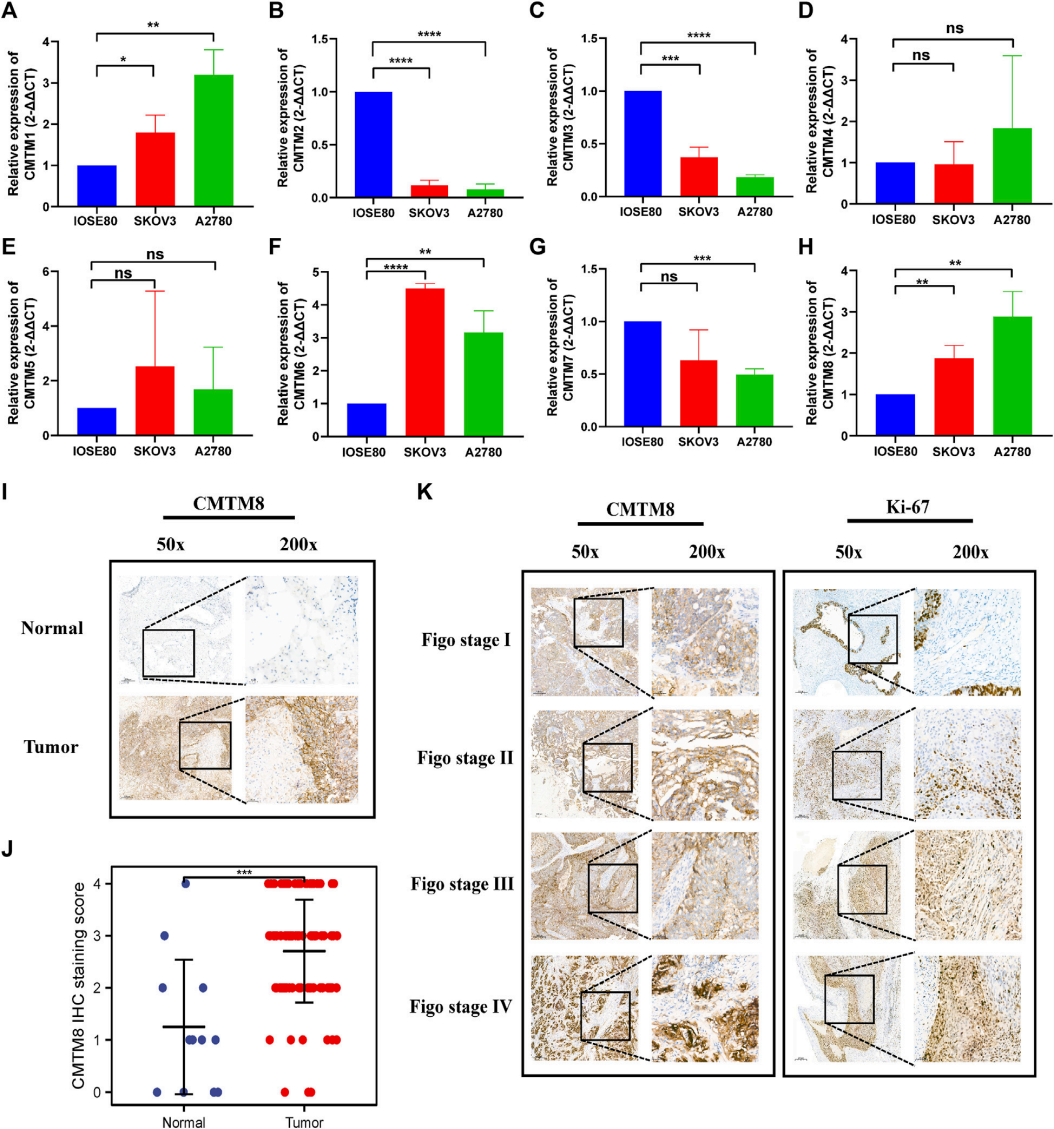
RT-qPCR and IHC was used to detect the expression of CMTM family genes in ovarian cancer and normal ovarian cell and tissues. **(A–H) RT-qPCR (A)** CMTM1. **(B)** CMTM2. **(C)** CMTM3. **(D)** CMTM4. **(E)** CMTM5. **(F)** CMTM6. **(G)** CMTM7. **(H)** CMTM8. **(I)** IHC of CMTM8 **(J)** Scatter plot of IHC staining score of CMTM8 **(K)** IHC staining of CMTM8 and immunohistochemistry of Ki-67 staining. (50x and 200x, *p < 0.05; **p < 0.01; ***p < 0.001; ns = Not statistically significant).

The first thing worth clarifying is the expression level of the CMTM gene family. Analysis of a total of 33 different types of tumors from the GEPIA database showed that CMTMs were expressed at different levels, suggesting that different members of the CMTM family play different roles in different cancer types, both as oncogenes and antioncogenes (Figure 1A). Among them, especially for ovarian cancer, CMTM3/4/6/7/8 had a relatively high expression in ovarian cancer compared to control normal tissues, while CMTM1/2/5 had a relatively low expression. Subsequently, analysis of the Oncomine database showed significant changes in the expression levels of CMTM3/7/8 mRNA in ovarian cancer tissues across multiple datasets (Figure 1B). In addition, the abnormal expression of the CMTM gene family was explored at the nucleic acid level and protein expression level, respectively. On the one hand, the differences in the expression of CMTMs were explored through 426 ovarian cancer tumor samples and 88 normal control samples from the GEPIA database. Figure 2A shows that the mRNA expression level of CMTM1/4/6/7/8 in tumors was relatively higher compared to normal control tissues, and the higher expression of CMTM6/7/8 was statistically significant, while the mRNA expression level of CMTM2/3/5 showed lower expression compared to normal controls. After exploring the nucleic acid levels, the differences in the expression of CMTMs at the protein level were explored with the help of immunohistochemistry data from the HPA database. As seen in Figure 2B, the protein expression of CMTM1/3/4/6 showed strong staining in ovarian cancer tissues. Finally, our RT-qPCR experiment confirmed that CMTM1/6/8 were higher expressed in ovarian cancer cell lines than in normal ovarian cells, but CMTM2/3/7 were lower expressed in ovarian cancer cell lines, and these differences are statistically significant (Figure 3). In addition, it was confirmed by immunohistochemistry that the CMTM8 gene is abnormally highly expressed in ovarian cancer tissues and is closely related to Ki-67 (Figures 3I–K). In conclusion, this study revealed that CMTMs were highly expressed in ovarian cancer tumor tissues at the mRNA level and the protein level, especially CMTM8, which indicated that they may be potential oncogenes. Therefore, further studies of CMTMs are needed to explore their impact or value in OV.

### Relationship between the mRNA expression of CMTM families and clinical characteristics of ovarian cancer

**FIGURE 4:**
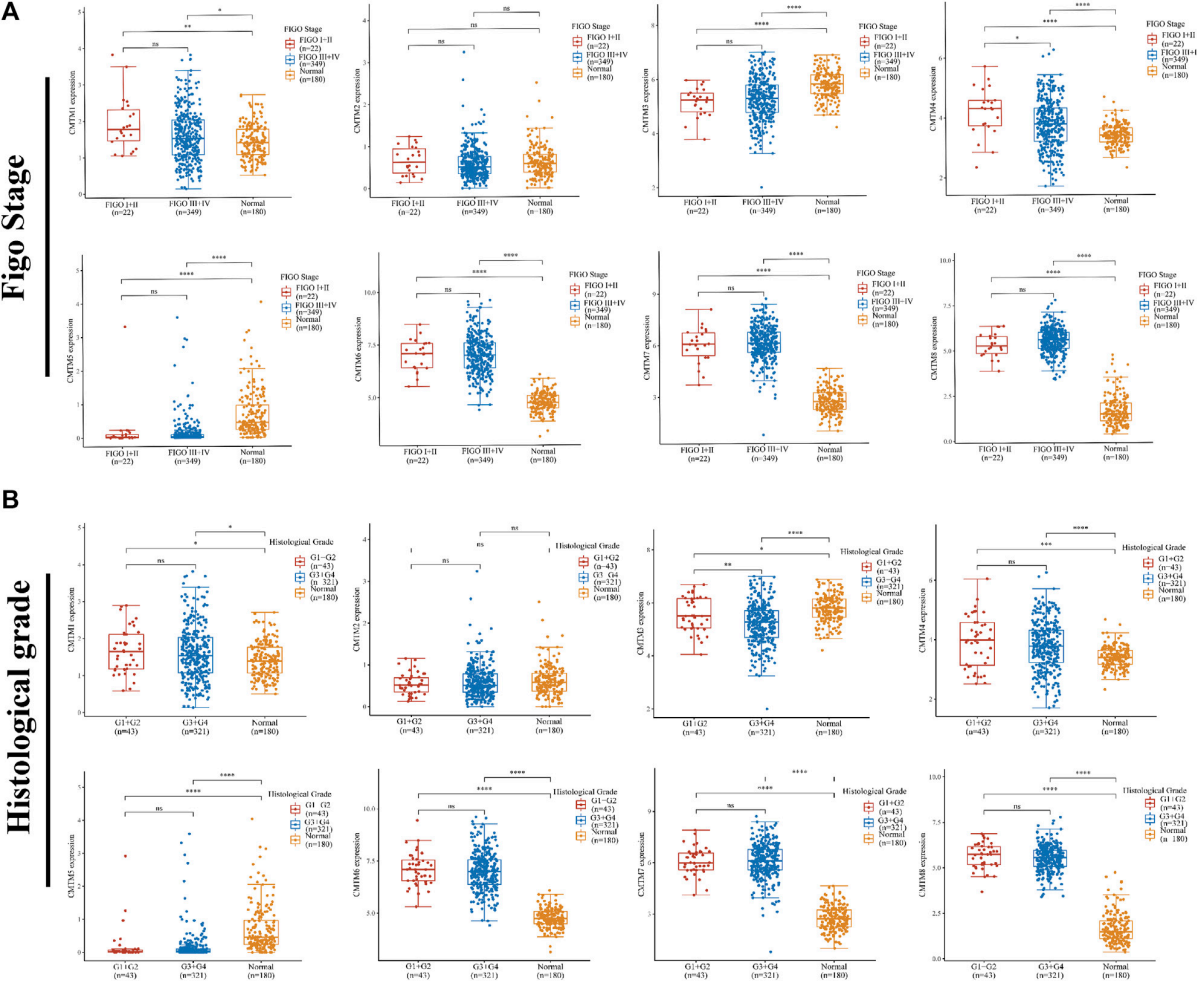
The relationship between the expression levels of each member of the CMTM family and different clinical characteristics. **(A)** Differences in mRNA expression levels of CMTMs in different FIGO stages (I + II = 22, III + IV = 349, normal = 180). **(B)** Differences in mRNA expression levels of CMTMs of different histological grades (G1+G2 = 43, G3+G4 = 321, normal = 180). *p < 0.05; **p < 0.01; ***p < 0.001.

Based on the abovementioned expression differences, the relationship between CMTM expression and clinical characteristics was further explored in a total of 371 ovarian cancer samples and 180 normal samples from the GEPIA database. First, there was a certain relationship between the expression level of the CMTM gene and FIGO staging. It can be seen from Figure 4A that the expression level of CMTM4/6/7/8 in patients with early FIGO stages (I and II) or late FIGO stages (III and IV) was significantly higher than that of normal control tissues. Second, there was also a certain relationship between the expression level of the CMTM gene and histological grade. It can be seen from Figure 4B that the expression level of CMTM4/6/7/8 in patients with early histological grade (G1 and G2) or late histological grade (G3 and G4) was significantly higher than that of normal control tissues. In conclusion, this study found that CMTMs were highly expressed in ovarian cancer tumor tissues in the TCGA database and were closely associated with important clinical factors such as FIGO stage and histological grade. Therefore, further studies on the prognosis of CMTMs are needed to explore their value for guidance and application in ovarian cancer clinics.

### Prognostic value of the CMTM gene family in ovarian cancer patients

**FIGURE 5:**
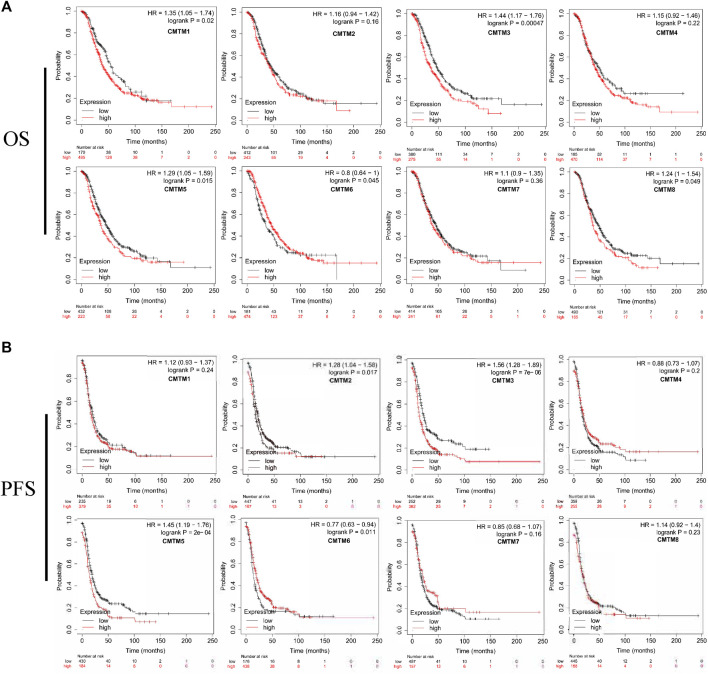
Survival analysis of ovarian cancer patients with high and low mRNA expression levels of different CMTM family members. **(A)** The survival curve of the overall survival time of the high gene expression group and the low gene expression group. **(B)** The survival curve of the progression-free survival of the high gene expression group and the low gene expression group. The logrank *p* value, HR (hazard ratio), and 95% confidence interval were recorded. *p* value of <0.05 was considered statistically significant.

In order to further explore the prognostic value of CMTMs for patients, the Kaplan–Meier Plotter database was used for survival analysis to evaluate the relationship between the expression level of CMTMs and OS or PFS. The results in Figure 5A show that the high expression of CMTM1/3/5/8 expression is related to the shorter overall survival of OV patients (*p* = 0.02, *p* = 0.00047, *p* = 0.015, *p* = 0.049, and HR > 1). As can be seen in Figure 5B, the high expression of CMTM2/3/5 is related to the shorter progression-free survival of OV patients (*p* = 0.017, *p* = 0.000007, *p* = 0.0002, and HR > 1). In general, it was not difficult to see that the abnormally high expression of CMTMs was a risk factor for the prognosis of OV patients.

### CMTM family gene alterations and its relationship with OS and DFS in patients with ovarian cancer

**FIGURE 6:**
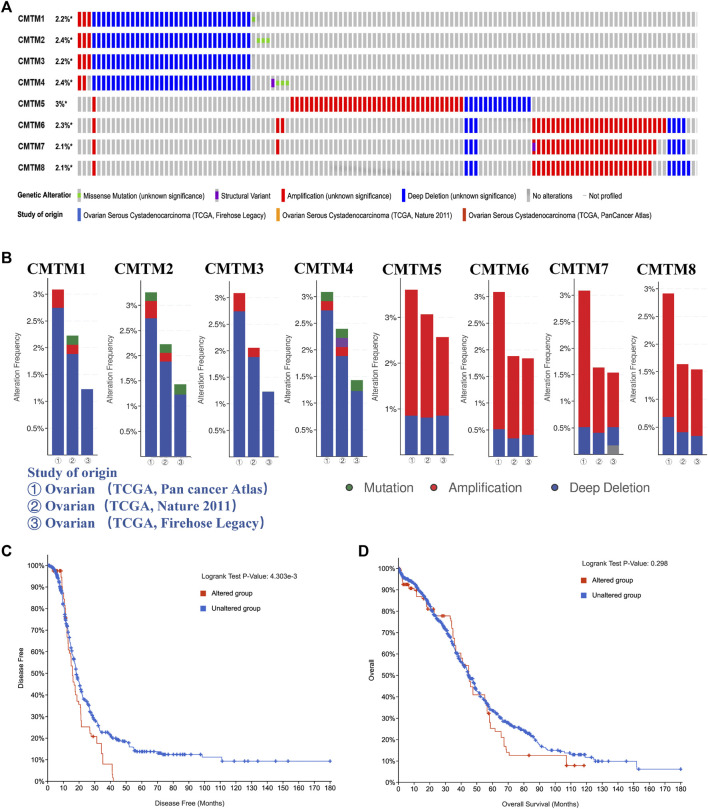
The genetic alteration of the members of the CMTM family in ovarian cancer and their effects on the prognosis. **(A)** Percentage of clinical samples with genetic alterations in ovarian cancer for each member of the CMTM family, including missense mutations, structural variants, amplifications, and deep deletions. For example, CMTM3 has genetic alterations in 2.2% of clinical samples, among which amplifications and deep deletions are the main ones, and the number of clinical samples with deep deletions is significantly higher than the number of clinical samples with amplifications. **(B)** Genetic alteration in three study cohorts, including TCGA PanCancer Atlas, TCGA Nature 2011 and TCGA Firehose Legacy. **(C)** Disease-free survival curves of ovarian cancer patients in the gene altered group and the gene unaltered group. **(D)** Survival curve of the overall survival time of ovarian cancer patients in the gene altered group and the gene unaltered group. *p* < 0.05 was considered statistically significant.

Genetic alterations play a crucial role in early malignancies. Through the analysis of the cBioPortal database, this study explored the genetic alterations of the CMTM gene family in patients with ovarian cancer, such as missense mutations, structural changes, amplifications, and deep deletions. Figure 6A showed that CMTM5/4/2/6 were the top four genes with genetic alterations, and their mutation rates were 3, 2.4, 2.4, and 2.3%, respectively. Figure 6B showed the details of the genetic alterations of the CMTM gene family in three cohorts (TCGA, Pan-cancer Atlas; TCGA, Nature 2011; and TCGA, Firehose Legacy). It can be seen from Figures 6A,B that the genetic alterations of the CMTM gene family for ovarian cancer were mainly amplification and deep deletion. Among them, CMTM1/2/3/4 were mainly deep deletions, while CMTM5/6/7/8 were mainly amplified, which corresponds to the abnormal overexpression of CMTMs found in the previous part of this study to some extent. In addition, the impact of genetic alterations on the survival of ovarian cancer patients was analyzed. Figures 6C,D showed that the disease-free survival of the altered group was significantly shortened (*p* = 0.004). In short, the occurrence and development of ovarian cancer may be related to genetic alterations in the CMTM family, and these alterations may lead to a poor prognosis for patients.

## 5 Analysis of GO and KEGG enrichment of CMTM family and co-expressed genes in patients with ovarian cancer

**FIGURE 7:**
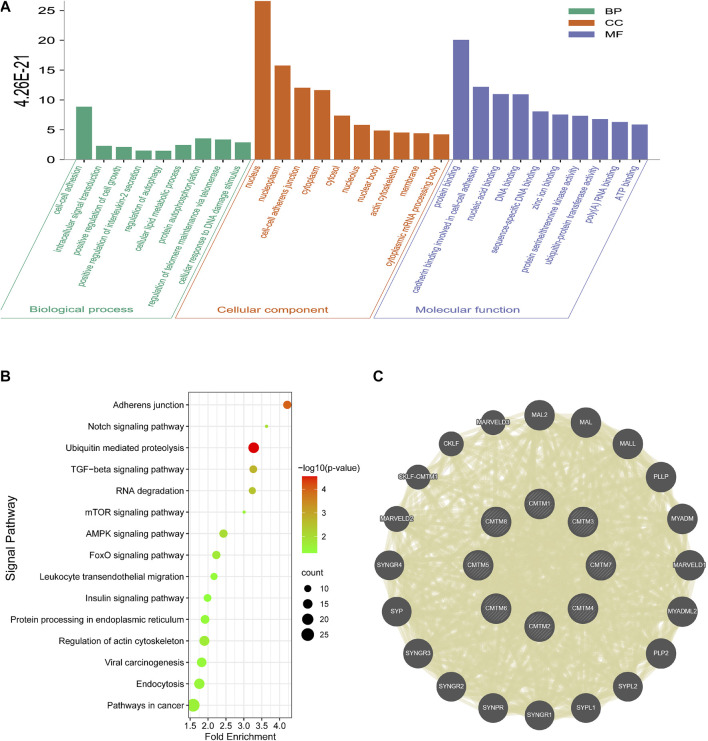
Go function annotation, KEGG pathway enrichment analysis and co-expression analysis of the CMTM family in OV. **(A)** The three components of Go functional annotation: biological processes, cellular components, and molecular functions. **(B)** Enrichment analysis of KEGG pathway. Enriched pathways included the AMPK signaling pathway, mTOR signaling pathway, Notch signaling pathway, and pathways in cancer. **(C)** Co-expressed genes of the CMTM family and their interaction network.

To further explore the potential molecular mechanisms of CMTMs in tumorigenesis of ovarian cancer, we sought to screen a series of pathways and biological functions through GO and KEGG. The results of the Go annotation analysis are shown in Figure 7A. Among them, the enriched biological processes included cell-cell adhesion, positive regulation of cell growth, regulation of autophagy, and regulation of telomere maintenance via telomerase. Also, the enriched molecular function included cadherin binding involved in cell-cell adhesion, protein binding, and nucleic acid binding. These biological processes and molecular functions may be closely related to malignant biological processes such as tumor cell proliferation, invasion, apoptosis, and autophagy. KEGG pathway enrichment analysis showed that the main enriched signaling pathways included mTOR signaling pathway, AMPK signaling pathway, and TGF-beta signaling pathway (Figure 7B). Finally, Figure 7C showed the top 20 genes that were co-expressed with members of the CMTM gene family. Among these twenty genes, there are genes closely related to immunity, such as MAL, MALL, and MAL2. For example, MAL2 inhibited tumor antigens to promote tumor immune escape and functions as an oncogene. This suggested that CMTMs, which are positively correlated with the expression of MAL2, may also function as oncogenes through the altered immune environment or immune process. Taken together, based on the analysis of biological processes, signal pathways, and co-expressed genes, we found that the CMTM gene family may be inseparably related to cell growth, cell adhesion, and immune regulation. Therefore, we ventured to speculate that the CMTM gene family may be able to promote the occurrence and development of ovarian cancer and lead to its poor prognosis by regulating cell growth, cell adhesion, and immune activity.

### The correlation of the CMTM gene family with immune infiltration and immune checkpoint and its prognosis

**FIGURE 8:**
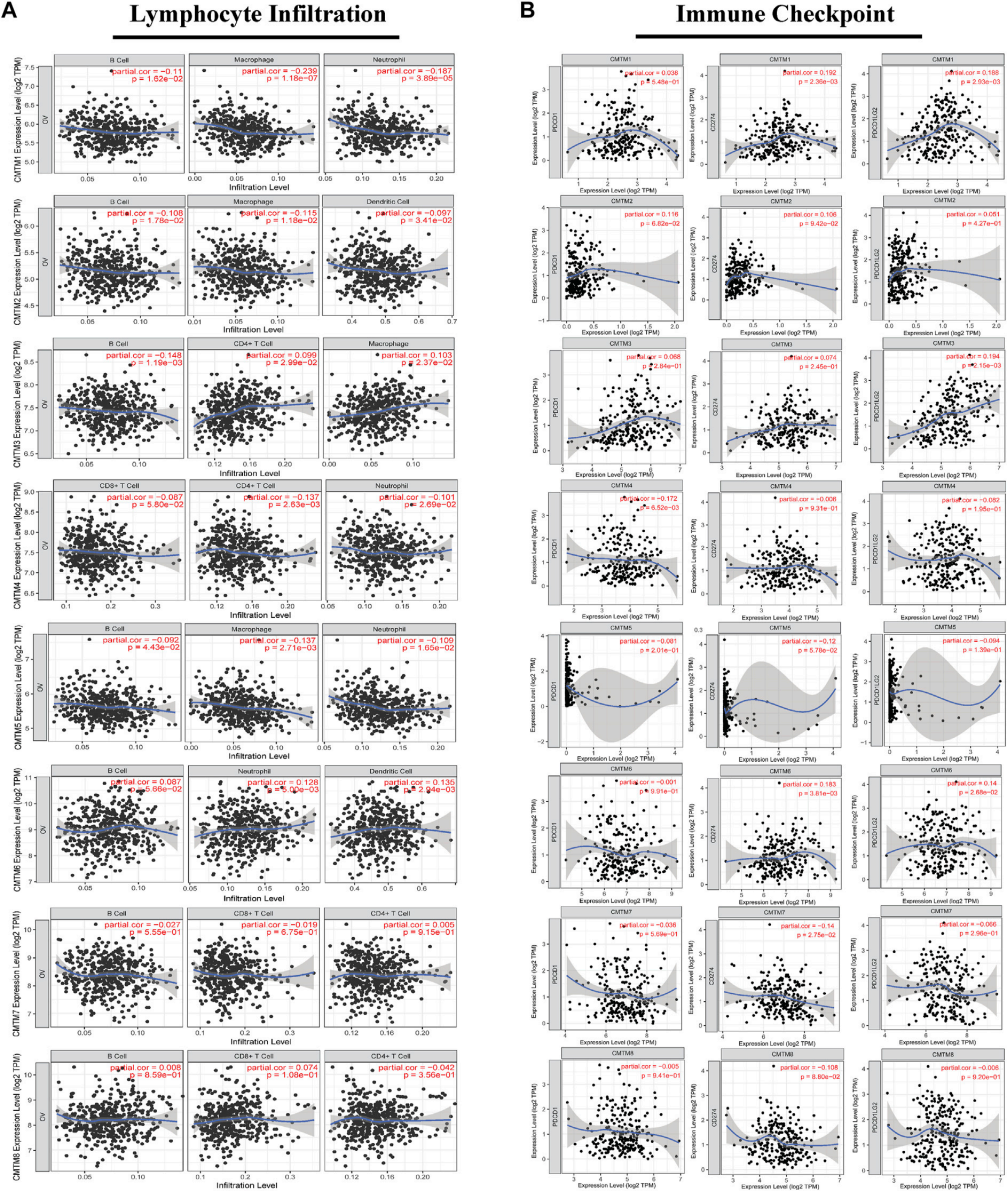
The correlation between the expression level of each member of the CMTM family and the degree of immune infiltration of multiple immune cells or the expression of immune checkpoint genes in ovarian cancer tissues. **(A)** The correlation between the expression level of each member of the CMTM family in ovarian cancer tissues and the degree of immune infiltration of a variety of immune cells, including B cells, CD8+T cells, CD4+T cells, macrophages, neutrophils, and dendritic cells. **(B)** The correlation between the expression level of CMTM and the genes PDCD1, CD274, and PDCD1LG2 that encode the immune checkpoints of PD1, PDL1, and PDL2. Spearman’s method was used for correlation analysis. Partial. cor >0 was considered a positive correlation. Partial. cor <0 was considered a negative correlation. *p* < 0.05 was considered statistically significant.

In this study, the TIMER database was selected to detect the relationship between CMTM expression and immune cell infiltration in ovarian cancer tissues. It could be seen that there was a certain correlation between the expression level of CMTMs and the degree of immune cell infiltration (Figure 8A). For example, the expression levels of CMTM1 and CMTM5 were positively correlated with the degree of infiltration of B cells, macrophages, and neutrophils (partial.cor >0, *p* < 0.05). The expression level of CMTM2 was positively correlated with the degree of infiltration of B cells, macrophages, and dendritic cells (partial.cor >0, *p* < 0.05). The expression level of CMTM3 was positively correlated with the degree of infiltration of B cells, macrophages and CD4^+^ cells (partial.cor >0, *p* < 0.05). The expression level of CMTM6 was negatively correlated with the degree of infiltration of B cells, neutrophils, and dendritic cells (partial.cor <0, *p* < 0.05).

**FIGURE 9:**
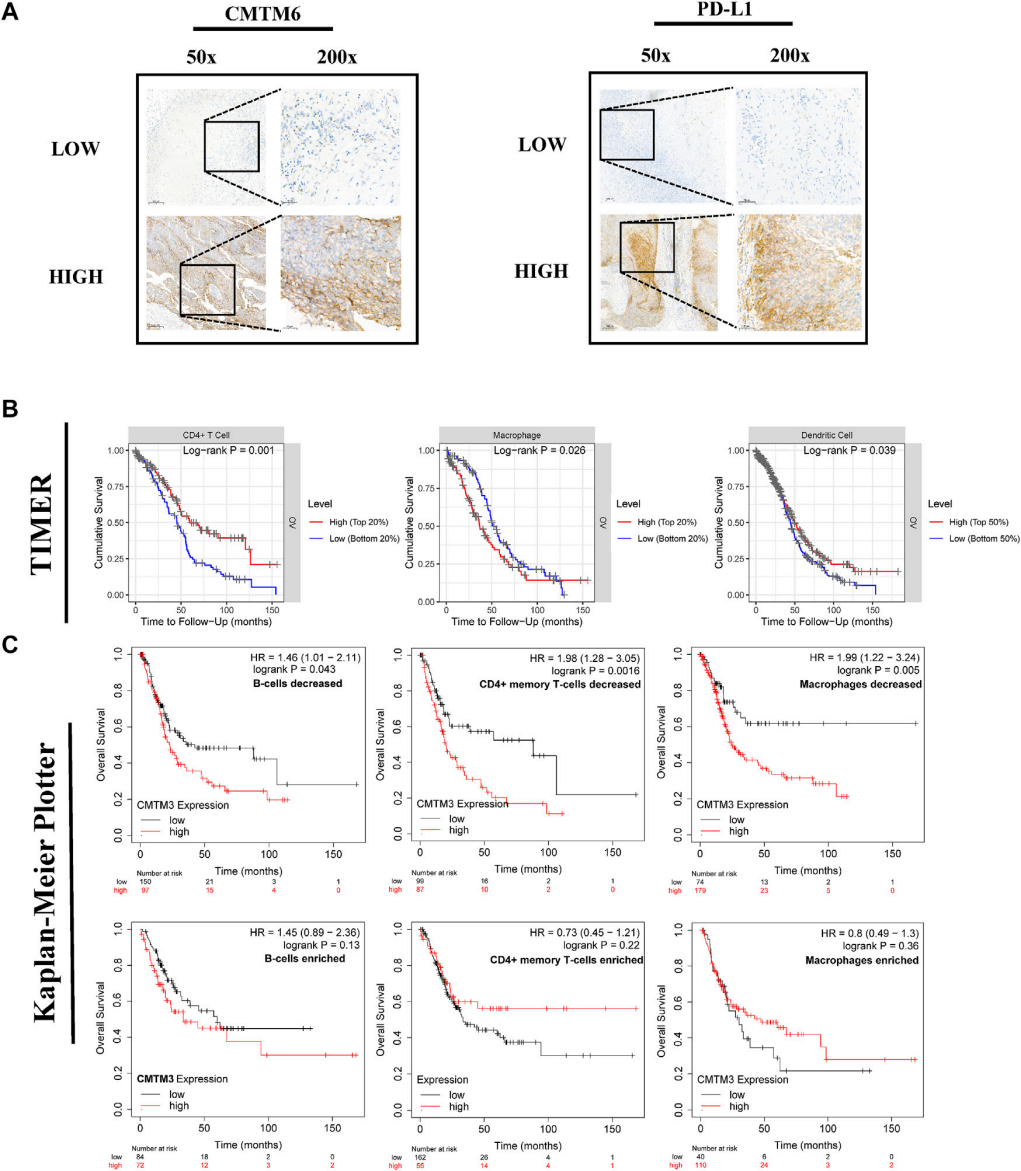
The immunohistochemical images of CD274 (PD-L1) and CMTM6, including 50x and 200x, respectively. The effect of CMTM3 expression level under different immune cell infiltration or different immune cell infiltration on the prognosis of patients with ovarian cancer. **(A)** The immunohistochemical images of CD274 (PD-L1) and CMTM6, including 50x and 200x, respectively. (HIGH represents the high-expression group of the CMTM6 gene; LOW represents the low-expression group of the CMTM gene) **(B)** Survival curves of different levels of immune cell infiltration, including dendritic cells, CD4^+^ cells, and macrophages. **(C)** Survival curves of the expression level of CMTM3 under different immune cell infiltrations, including B cells, CD4^+^ cells, and macrophages. The logrank *p* value, HR (hazard ratio), and 95% confidence interval were recorded. *p* value of <0.05 was considered statistically significant.

At the same time, numerous studies have confirmed that immune checkpoints promote the occurrence and development of cancer to some extent by changing the immune process, and treatment methods that suppress immune checkpoints and their receptors or ligands are promising therapeutic options for the induction of effective anticancer immunity. Therefore, we analyzed the correlation between the expression level of CMTM and the genes PDCD1, CD274, and PDCD1LG2 that encode the immune checkpoints of PD1, PD-L1, and PD-L2 (Figure 8B). The results showed that the expression level of CMTM1/2/3 was positively correlated with the gene expression levels of PDCD1, CD274, and PDCDLG2 (partial.cor >0, *p* < 0.05). Also, the expression level of CMTM6 was positively correlated with the gene expression levels of CD274 and PDCDLG2 (partial.cor >0, *p* < 0.05). Subsequently, we confirmed by immunohistochemistry that CMTM6 is significantly positively correlated with the CD274 (PD-L1) gene, and the expression of CD274 (PD-L1) in the high expression group of the CMTM6 gene is also abnormally increased (Figure 9A). As mentioned previously, In general, the expression level of the CMTM family was closely related to the level of immune infiltration and immune checkpoints that play an important role in the occurrence and development of ovarian cancer.

The previous part of this study showed that the expression level of CMTMs has an impact on the poor prognosis of OV patients (Figure 5) and was related to immune cell infiltration (Figure 8A). Therefore, the impact of immune cell infiltration on survival was further explored (Figure 9B). The results showed that the different levels of infiltration of dendritic cells, CD4^+^ cells, and macrophages could lead to shorter survival times for patients with ovarian cancer (*p* < 0.05). In addition, this study also deeply explored the impact of the CMTM family gene expression level represented by CMTM3 on the survival of ovarian cancer patients under different immune cell infiltration (B cells, CD4^+^ T cells, and macrophages) (Figure 9C). The results showed that under these three immune cell infiltration conditions, the OS of the high expression group of CMTM3 was significantly shorter than that of the low expression group (*p* < 0.05).

In conclusion, the immune correlation analysis results based on the TIMER database and the Kaplan-Meier plotter database showed that the CMTM family was closely related to immune cell infiltration and immune checkpoints and may lead to poor survival of ovarian cancer patients by regulating these two important parts.

## Discussion

Although, with the advent of the “big data” era and the rapid development of precision medicine, the molecular characteristics of ovarian cancer that are considered immunogenic tumors are still not completely clear. Therefore, there is an urgent need to continue to explore specific genes related to immunity with high target specificity and high sensitivity that can be used as targets for OV treatment and to evaluate treatment response and prognosis (Gomes et al., 2019). We were surprised to find that most members of the CMTM family of proteins have been reported to have specific roles in immunobiology. Yet, the role of this gene family in ovarian cancer has not been revealed. Therefore, this study provided the first more comprehensive analysis of the CMTM gene family in OV in terms of gene expression, genetic alterations, prognostic value, potential molecular mechanisms, and changes in the immune microenvironment. The mRNA expression level of CMTM gene family in ovarian cancer cell lines has also been experimentally verified.

First of all, the expression level of each member of the CMTM family in pan-cancer has been explored. As shown in Figures 1A,B, CMTMs were found to be significantly abnormally expressed in a variety of malignancies using the GEPIA database and ONCOMINE database. Also, some studies have reported that CMTM5 and CMTM6 are closely related to ovarian cancer immune cell infiltration as biomarkers for ovarian cancer prognosis (Li et al., 2011; Yin et al., 2022). In view of the above pan-cancer results, we sought to validate the expression levels of CMTMs in OV. Then, the GEPIA database was used to investigate the differences in mRNA expression levels of CMTMs in ovarian cancer tissues and normal control ovarian tissues, and the results showed that CMTM6/7/8 were abnormally highly expressed in ovarian cancer tissues (Figure 2A). In addition, this study also found that CMTM1/3/4/6 exhibited abnormally high expression at the protein level in the HPA database, as shown in Figure 2B. Numerous studies have confirmed that CMTM1-8 presents aberrant overexpression or under-expression in a variety of tumors. Subsequently, we validated and explored the expression of CMTM family genes in ovarian cancer cell lines (SK-OV-3 and A2780) and normal ovarian cell lines (IOSE80) using RT-qPCR. Interestingly, we found that CMTM1/6/8 was aberrantly highly expressed in ovarian cancer cell lines (Figure 3), which is highly consistent with the results of the previous database analysis of this study. Surprisingly, through IHC experiments, the CMTM8 gene is abnormally highly expressed in ovarian cancer tissues compared with normal ovarian tissues. In addition, the staining intensity of CMTM8 increases in advanced ovarian cancer, and it is closely related to the staining of Ki-67 (Figures 3I–K). The abovementioned results indicated that multiple members of the CMTM gene family are higher expressed or lower expressed in ovarian cancer, suggesting that CMTMs are a potential oncogene or oncogene suppressor playing a role in ovarian cancer. In particular, CMTM8 may be related to the malignant proliferation and late clinical staging of ovarian cancer in addition to being an oncogene of ovarian cancer.

The CMTM gene family has prognostic value in a variety of cancers including lung cancer, gastric cancer, and glioma. Given the above phenomena, we are interested in whether CMTMs may contribute to the poor prognosis of OV patients. First, the correlation analysis of clinical characteristics found that the expression levels of multiple members of the CMTM family were significantly correlated with Figo Stage and Histological grade, indicating that the CMTM gene family was related to the poor prognosis of ovarian cancer, and these two clinical characteristics were recognized as factors related to the prognosis of ovarian cancer (Figures 4A,B). Importantly, in the current study, we showed evidence of an association between CMTM1/3/5/8 and the overall survival of ovarian cancer patients (Figure 5A). In addition, CMTM2/3/5 and progression-free survival of ovarian cancer patients were significantly associated (Figure 5B). Therefore, the abnormally high expression of CMTM3/5 and other family members was related to prognostic-related clinical characteristics and can affect the overall survival and progression-free survival of patients with ovarian cancer, indicating that it can be used as an influencing factor for the prognosis of patients with ovarian cancer. However, the exact mechanism by which this gene family affected the prognosis of patients with ovarian cancer was still unclear, and further exploration and research were needed.

A growing body of *ex vivo* and *in vivo* evidence suggested that the mechanism of action of most CMTM family members is complex and multifactorial. To further understand the detailed mechanism of the CMTM gene family leading to the poor prognosis of OV, first, we explored the genetic alterations of the CMTM gene family through cBioportal. Frequent and diverse genetic alterations were seen in CMTMs, and patients with genetic alterations in this gene family had a shorter overall survival in ovarian cancer (Figures 6A–D). Second, GO annotation analysis and KEGG analysis showed a close correlation between CMTMs and malignant biological processes such as tumor cell proliferation, apoptosis, and immune escape. (Figures 7A,B). Also, the TGF-β signaling pathway enriched in the KEGG analysis is a key executor of immune balance and tolerance, inhibiting the function of the immune system (Batlle and Massague, 2019). It is worth noting that studies have reported that the TGF-β pathway has a significant impact on the prognosis and immune escape of ovarian cancer patients (Roane et al., 2019). Studies have also reported that FOXOs promoted anti-tumor activity by silencing the expression of immunosuppressive proteins, such as inhibiting the expression of Programmed cell death 1 ligand 1 (PD-L1) to cause the immune escape phenomenon of tumor cells (Deng et al., 2018). Finally, among the genes co-expressed with CMTMs, studies have reported that MAL2, which was positively correlated with the expression of CMTMs, can act as an oncogene to inhibit tumor antigens and promote tumor immune escape (Fang et al., 2021). This indicated that the CMTM family may also change the immune microenvironment and regulate the malignant process of ovarian cancer by co-expressed genes. From the above, it was clear that the CMTM gene family was enriched in immune-related signaling pathways, thereby regulating the immune microenvironment and playing a role in ovarian cancer.

The change of tumor microenvironment (TME) has become the focus of cancer mechanism research. For instance, the immunosuppressive tumor microenvironment enables tumor cells to escape the body’s immune response, and disables the body’s anti-tumor mechanism, thereby increasing the occurrence and development of various tumors (Guan et al., 2018). The analysis of the immune microenvironment in this study showed that the expression level of CMTM1/2/3/6 had a certain correlation with the infiltration of a variety of immune cells (B cells, macrophages, neutrophils, dendritic cells, and CD4^+^ cells) in ovarian cancer (Figure 8A). Also, immune infiltration has been proven to play a decisive role in the pathogenesis and prognosis of a variety of malignant tumors, including ovarian cancer (Santoiemma and Powell, 2015). In addition, there was a positive correlation between the expression level of CMTM1/2/3/6 and the genes PDCD1, CD274, and PDCD1LG2 that encode PD1, PD-L1, and PD-L2 immune checkpoints (Figure 8B). These immune checkpoints have been widely considered to be related to the immune escape of tumor cells and can promote the progression of ovarian cancer, and treatment options for these immune checkpoints, such as anti-PD-L1 therapy, can significantly improve the prognosis of patients with recurrent ovarian cancer. Also, CMTM6 was the first CMTM family member associated with tumor immune escape, which regulated endocytic recycling and degradation of PD-L1. The results of our immunohistochemical experiments also confirmed this relationship. CD274 (PD-L1) in the CMTM6 gene high expression group also showed abnormally high expression (Figure 9A). In addition, CMTM4 can act as an alternative to CMTM6 in case of malfunction to exert immunosuppressive effects. As mentioned above, the expression level of multiple members of CMTMs, such as CMTM1/2/3/6, was closely related to the level of immune cell infiltration and multiple immune checkpoints and may lead to the poor prognosis of ovarian cancer through related immunological mechanisms.

This study had certain advantages. For example, for the first time, this study analyzed the members of the CMTM gene family as a whole in ovarian cancer. In addition, this study explored the expression level of the CMTM family in ovarian cancer and its prognostic evaluation based on the large sample size of multiple databases and tried to explore its potential mechanism of action through genetic alterations and immune microenvironment. Also, more importantly, this study performed RT-qPCR and IHC on the corresponding cell lines and tissues, which strongly verified the expression of CMTM in ovarian cancer and greatly improved the scientific nature of this study. However, this research still had certain flaws inevitably. On the one hand, multiple public databases were used in this study. Although we tried to normalize and standardize these data, there was inevitably certain background heterogeneity. On the other hand, in exploring the detailed mechanism in which CMTM may participate, this study only made scientific and reasonable predictions as far as possible in order to point out the direction and pave the way for the follow-up research, but the specific situation needs to be verified in detail.

## Conclusion

This study showed that CMTM family members such as CMTM1/2/3/6/8 had abnormal expression in ovarian cancer and could cause poor prognosis. Moreover, the CMTM family was related to immune cell infiltration and immune checkpoints and led to changes in the immune microenvironment, indicating that the CMTM family may be used as a potential target for immune checkpoint-based immunotherapy. The most important thing is that we found that CMTM8 can be used as an oncogene for ovarian cancer and may be related to its malignant proliferation. The most exciting thing is that we found that CMTM6 is closely related to CD274 (PD-L1) and may be used as a target for immunotherapy.

## Data Availability

The datasets presented in this study can be found in online repositories. The names of the repository/repositories and accession number(s) can be found in the article/Supplementary Material.

## Abbreviations

CI, Confidence interval; FDR, False discovery rate; GEPIA, Gene Expression Profiling Interactive Analysis; HPA, Human Protein Atlas; HR, Hazard ratio; IHC, Immunohistochemistry; KEGG, Kyoto Encyclopedia of Genes and Genomes; NO, M nominal; OS, Overall survival; OV, Ovarian cancer; RT-qPCR, Real-time quantitative polymerase chain reaction; TCGA, The Cancer Genome Atlas; TIMER, Tumor Immune Estimation Resource
