# Supplementary material for: Identification of prognostic biomarkers in the CMTM family genes of human ovarian cancer through bioinformatics analysis and experimental verification
Source: Front Genet. 2022 Aug 30;13:918319. doi: 10.3389/fgene.2022.918319 (PMC9468640; doi:10.3389/fgene.2022.918319)
Supplement: Supplementary file 1 [file Table1.docx]

**Table S1. CMTM Gene Family summary table.**

| **Gene** | **mRNA expression**  **(Normal VS Tumor)** | **Protein expression**  **(Normal VS Tumor)** | **Prognosis**  **(mRNA expression /OS)** | **Immune correlation** | |  | **Research progress** | |
| --- | --- | --- | --- | --- | --- | --- | --- | --- |
|  |  |  |  | **Lymphocyte infiltration** | **Immune checkpoint** |  |  |  |
| **CMTM1** | **n.s** | **Upregulated** | **Negative** | **B cell；Marcrophage；Neutrophil** | **PD-L1; PD-L2** | **Lung cancer^1^；Breast cancer^2^；Glioblastoma^3^**  **Hepatocellular carcinoma^4^；** | | |
| **CMTM2** | **n.s** | **n.s** | **n.s** | **B cell；Marcrophage；Dendritic** | **n.s** | **Hepatocellular Carcinoma^5^；** | | |
| **CMTM3** | **n.s** | **Upregulated** | **Negative** | **B cell；CD4+T cell；Marcrophage** | **PD-L2** | **Colorectal cancer^6^；Gastric cancer^7^；Testicular cancer^8^；Prostate cancer^9^；Hepatocellular Carcinoma^10^；** | | |
| **CMTM4** | **n.s** | **Upregulated** | **n.s** | **CD4+T cell；Neutrophil** | **PD-1** | **Colorectal cancer^11^；Renal cell carcinoma^12^；** | | |
| **CMTM5** | **n.s** | **n.s** | **Negative** | **B cell；Marcrophage；Neutrophil** | **n.s** | **Prostate cancer^13^；Renal cancer^14^；Pancreatic cancer^15^;** **Breast cancer^16^;** **Ovarian cancer^17^;** | | |
| **CMTM6** | **Upregulated** | **Upregulated** | **Negative** | **B cell；Marcrophage；Dendritic** | **PD-L1; PD-L2** | **Lung Cancer^18^;** **Head and Neck Squamous Cell Carcinoma^19^;** **Gastric cancer^20^;** **Gliomas^21^;** **Hepatocellular Carcinoma^22^;** | | |
| **CMTM7** | **Upregulated** | **n.s** | **n.s** | **n.s** | **PD-L1** | **Liver cancer^23^; Lung cancer^24^;** **Gastric carcinoma^25^;** **Breast Cancer^26^;** | | |
| **CMTM8** | **Upregulated** | **n.s** | **Negative** | **n.s** | **n.s** | **Bladder cancer^27^; Lung Cancer^28^;** **Renal cell carcinoma^29^;** **Gastric Cancer^30^;** | | |

**参考文献：**

**1.** **CMTM1_v17 is associated with chemotherapy resistance and poor prognosis in non-small cell lung cancer.**

**2.** **CMTM1_v17 is a novel potential therapeutic target in breast cancer**

**3.** **Systematic investigation of CMTM family genes suggests relevance to glioblastoma pathogenesis and CMTM1 and CMTM3 as priority targets**

**4.** **Expression and clinical significance of CMTM1 in hepatocellular carcinoma**

**5.** **Downregulated CMTM2 Poses Potential Clinical Significance in Hepatocellular Carcinoma**

**6.** **DNA methylation of CMTM3, SSTR2, and MDFI genes in colorectal cancer**

**7.** **CMTM3 inhibits cell migration and invasion and correlates with favorable prognosis in gastric cancer**

**8.** **CMTM3 inhibits human testicular cancer cell growth through inducing cell-cycle arrest and apoptosis**

**9.** **CMTM3 is reduced in prostate cancer and inhibits migration, invasion and growth of LNCaP cells**

**10.** **CKLF-Like MARVEL Transmembrane Domain-Containing Member 3 (CMTM3) Inhibits the Proliferation and Tumorigenisis in Hepatocellular Carcinoma Cells**

**11.** **CMTM4 inhibits cell proliferation and migration via AKT, ERK1/2, and STAT3 pathway in colorectal cancer**

**12.** **CMTM4 is frequently downregulated and functions as a tumour suppressor in clear cell renal cell carcinoma**

**13.** **Expression and significance of CMTM5 and epidermal growth factor receptor in prostate cancer**

**14.** **CMTM5 inhibits renal cancer cell growth through inducing cell-cycle arrest and apoptosis**

**15.** **CMTM5 induces apoptosis of pancreatic cancer cells and has synergistic effects with TNF-alpha**

**16.** **Bioinformatics-Based Discovery of CKLF-Like MARVEL Transmembrane Member 5 as a Novel Biomarker for Breast Cancer 17.**

**17.** **Reduced CMTM5 expression correlates with carcinogenesis in human epithelial ovarian cancer**

**18.** **Quantitative Assessment of CMTM6 in the Tumor Microenvironment and Association with Response to PD-1 Pathway Blockade in Advanced-Stage Non-Small Cell Lung Cancer**

**19.** **Targeting CMTM6 Suppresses Stem Cell-Like Properties and Enhances Antitumor Immunity in Head and Neck Squamous Cell Carcinoma**

**20.** **CMTM6 significantly relates to PD-L1 and predicts the prognosis of gastric cancer patients**

**21.** **CMTM6 overexpression is associated with molecular and clinical characteristics of malignancy and predicts poor prognosis in gliomas**

**22.** **Expression and Clinical Significance of CMTM6 in Hepatocellular Carcinoma**

**23.** **Overexpression of CMTM7 inhibits cell growth and migration in liver cancer**

**24.** **CMTM7 knockdown increases tumorigenicity of human non-small cell lung cancer cells and EGFR-AKT signaling by reducing Rab5 activation**

**25.** **SOX10-dependent CMTM7 expression inhibits cell proliferation and tumor growth in gastric carcinoma**

**26.** **Epithelial to Mesenchymal Transition Regulates Surface PD-L1 via CMTM6 and CMTM7 Induction in Breast Cancer**

**27.** **CMTM8 inhibits the carcinogenesis and progression of bladder cancer**

**28.** **Research Advances in CKLF-like MARVEL Transmembrane Domain-containing Family in Non-small Cell Lung Cancer**

**29.Expressions of CMTM8 and E-cadherin in primary and metastatic clear cell renal cell carcinoma**

**30.** **Downregulated CMTM8 Correlates with Poor Prognosis in Gastric Cancer Patients**
