# Supplementary material for: Identification of prognostic biomarkers in the CMTM family genes of human ovarian cancer through bioinformatics analysis and experimental verification
Source: Front Genet. 2022 Aug 30;13:918319. doi: 10.3389/fgene.2022.918319 (PMC9468640; doi:10.3389/fgene.2022.918319)
Supplement: Supplementary file 2 [file Table2.docx]

**Table S2. Characteristics of patients with OV based on clinical samples**

| Characteristics |  | Number of cases |
| --- | --- | --- |
| Age | <=60 | 73 |
|  | >60 | 25 |
| Clinical stage | Stage I-II | 8 |
|  | Stage III-IV | 90 |
| Lymph node involvement | NO | 66 |
|  | YES | 32 |
| Histologic grade | G1-G2 | 27 |
|  | G3 | 71 |
| Tumor residual disease | 0 mm | 82 |
|  | ＞0 mm | 16 |
| Ascites | NO | 51 |
|  | YES | 47 |
| CMTM8 expression level | HIGH | 87 |
|  | LOW | 11 |
